# Association of Sperm Methylation at *LINE-1*, Four Candidate Genes, and Nicotine/Alcohol Exposure With the Risk of Infertility

**DOI:** 10.3389/fgene.2019.01001

**Published:** 2019-10-18

**Authors:** Wenjing Zhang, Min Li, Feng Sun, Xuting Xu, Zhaofeng Zhang, Junwei Liu, Xiaowei Sun, Aiping Zhang, Yupei Shen, Jianhua Xu, Maohua Miao, Bin Wu, Yao Yuan, Xianliang Huang, Huijuan Shi, Jing Du

**Affiliations:** ^1^NHC Key Laboratory of Reproduction Regulation (Shanghai Institute of Planned Parenthood Research), Fudan University, Shanghai, China; ^2^Reproductive Medical Center, Changhai Hospital of Shanghai, Shanghai, China; ^3^Department of Obstetrics and Gynecology, International Peace Maternity and Child Health Hospital, Shanghai Jiao Tong University, Shanghai, China; ^4^Huzhou Key Laboratory of Molecular Medicine, Huzhou Central Hospital, Zhejiang, China; ^5^Bio-X Institutes, Key Laboratory for the Genetics of Developmental and Neuropsychiatric Disorders, Ministry of Education, Shanghai Jiao Tong University, Shanghai, China; ^6^Shanghai Institute of Planned Parenthood Research Hospital, Shanghai, China

**Keywords:** DNA methylation, nicotine/alcohol exposure, male infertility, imprint gene, sperm

## Abstract

In this study, we examined whether smoking and drinking affect sperm quality and the DNA methylation of the repetitive element *LINE-1*, *MEST*, *P16*, *H19*, and *GNAS* in sperm. Semen samples were obtained from 143 male residents in a minority-inhabited district of Guizhou province in southwest China. Quantitative DNA methylation analysis of the samples was performed using MassARRAY EpiTYPER assays. Sperm motility was significantly lower in both the nicotine-exposed (P = 0.0064) and the nicotine- and alcohol-exposed (P = 0.0008) groups. Follicle-stimulating hormone (FSH) levels were higher in the nicotine-exposed group (P = 0.0026). The repetitive element *LINE-1* was hypermethylated in the three exposed groups, while *P16* was hypomethylated in the alcohol and both the alcohol and nicotine exposure groups. Our results also show that alcohol and nicotine exposure altered sperm cell quality, which may be related to the methylation levels of *MEST* and *GNAS*. In addition, *MEST*, *GNAS*, and the repetitive element *LINE1* methylation was significantly associated with the concentration of sperm as well as FSH and luteinizing hormone levels.

## Introduction

Infertility is a major public health concern that affects 10%–20% of married couples attempting to conceive, and male infertility is the only or a common factor (Sharlip et al., 2002; Dada et al., 2008). Recent epidemiological studies have provided plenty of pieces of evidence that environmental exposure, lifestyle, and DNA methylation are closely related to male infertility (Kobayashi et al., 2017; Laqqan et al., 2017; Nasri et al., 2017; Santi et al., 2017; Donkin and Barres, 2018; Siddeek et al., 2018). Our studies before had shown associations of aberrant DNA methylation of several genes in spermatozoa with male infertility, but the study before chose only an asthenozoospermia patient, and other environmental elements such as smoking and drinking were not examined (Xu et al., 2016). It has been shown that long-term alcohol consumption and tobacco use have adverse effects on fertility (Besingi and Johansson, 2014; Hamad et al., 2018). Tobacco exposure is associated with impaired fecundability (Chavarro et al., 2009), slightly lower sperm viability, and reduced ejaculate volume, and spermatocyte apoptosis and disruption of the seminiferous tubules were observed (Kunzle et al., 2003; Gunes et al., 2018). Although cigarette smoking and alcohol consumption have been shown to affect DNA methylation patterns of human sperm, be related to semen quality, and have effects on endocrine control of reproductive and sexual functions, their effects on semen parameters is controversial (Gaur et al., 2010; Hansen et al., 2012; Povey et al., 2012; Chang et al., 2017; Laqqan et al., 2017; Alkhaled et al., 2018; Al Khaled et al., 2018).

Several studies have shown that smoking and drinking have similar effects on oxidative stress and DNA methylation in males of reproductive age and animal models, as has been observed for other chemicals, such as cadmium and bisphenol A (BPA) (Besingi and Johansson, 2014; Miao et al., 2014; Pierron et al., 2014; Jenkins et al., 2017; McCarthy et al., 2018). In imprinted genes, the methylation level present at pro-meiosis should be maintained throughout the male gamete development (Yaman and Grandjean, 2006; Marques et al., 2011). Nicotine exposure may alter the methylation of imprinted and non-imprinted genes in sperm that are associated with oligozoospermia and asthenozoospermia (Dai et al., 2017; Dong et al., 2017; Laqqan et al., 2017). Studies have also shown that chronic paternal alcohol exposure induced behavioral abnormalities in offspring due to alterations in the methylation of imprinted genes in sperm (Kim et al., 2014; Liang et al., 2014; Chastain and Sarkar, 2017).

Mesoderm-specific transcript (*MEST*) and *GNAS* are two maternally imprinted genes that are expressed from the paternal allele. The germ-line differentially methylated regions (DMRs) in *MEST* and *GNAS* exhibit differences in methylation levels between sperm and egg. During spermatogenesis, sperm genomic imprinting (especially in germ-line DMRs) is vulnerable to environmental factors (Marques et al., 2008). In addition, alcohol exposure could cause hypomethylation of *H19* in the sperm of offspring and reduce the mean sperm concentration (Marques et al., 2011), and the methylation levels of *H19* are related to sperm parameters, sperm chromatin, and DNA integrity (Montjean et al., 2015; Darbandi et al., 2018).

The promoter of long interspersed nucleotide element (*LINE-1*), which is used as a surrogate for global methylation, is enriched with methylated CpG dinucleotides and is usually silenced in normal tissues (Rangwala et al., 2009). However, the methylation levels of the *LINE-1* DMRs were lower in BPA-exposed spermatozoa and asthenozoospermia (Miao et al., 2014; Xu et al., 2016). In addition, the P16 protein may inhibit mitosis in spermatogonia and is related to a loss of testicular function (Xin-Chang et al., 2002; Jeong et al., 2017), and we showed that increased methylation defects in the *P16* DMR may be associated with low sperm motility (Xu et al., 2016). The imprint and non-imprint methylation marks at these DMRs are established during gametogenesis and affected by environmental exposures (Li et al., 2016). *P16* methylation is strongly associated with smoking in different pathological conditions, including lung cancer and cervical cancer (Han et al., 2016; Han et al., 2017; Wang et al., 2017). However, the relationship between these genes, tobacco/alcohol exposure, and male infertility has not yet been elucidated.

To investigate the methylation modifications that occur under exposure to alcohol and nicotine, we performed a cohort study of the methylation at the repetitive element *LINE-1* and four genes (*MEST*, *P16*, *H19*, and *GNAS*) in 143 subjects. The aim of this study was to assess whether the DNA methylation of these five genes is associated with the risk of male infertility under tobacco/alcohol exposure.

## Methods

### Subjects and Clinical Data

This study included 143 male residents from a minority-inhabited district in Sandu county of Guizhou province. All participants were interviewed by trained Chinese-speaking researchers and were asked about their demography, disease history, and lifestyle factors, including tobacco use and alcohol consumption. The standards for smokers and drinkers were as we described before (Liang et al., 2017; Yang et al., 2017). We defined smoking as consuming at least one cigarette per day for more than 6 months and drinking as consuming an alcohol beverage (beer, wine, and liquor) at least once a week for more than 6 months (Witkiewitz et al., 2017; Pang et al., 2018). As per the standards of the World Health Organization, semen samples were collected after 2 days of abstinence. Sperm counts and motility were assessed by a computer-aided sperm analysis system (Cyto-S; Alpha Innotech Corp., San Leandro, CA, USA) at 37°C. The remainder of the semen sample was stored at −80°C until further examination and DNA extraction. Our study was approved by the Ethics Committee of Shanghai Institute of Planned Parenthood Research, and the local approval of Guizhou province was not required. The individuals included in this study gave written informed consent before participating. All procedures were carried out in accordance with the approved guidelines and local regulations.

### DNA Methylation Assay

DNA was extracted from the semen samples by using the QIAamp DNA Mini Kit (Qiagen, Valencia, CA, USA) stored at −80°C. Bisulfite conversion of DNA was carried out using the Epitect Bisulfite Kit (Qiagen). Quantitative analysis of DNA methylation was performed using MassARRAY EpiTYPER assays (Sequenom, San Diego, CA, USA) according to a published protocol (He et al., 2013). Primers used in this study were designed using Methprimer (http://epidesigner.com; **Table 1**). CpG units that yielded data in >90% of the samples passed the initial quality control assessment. Epigenetic changes at DMRs are important in controlling the levels of gene expression. Thus methylation was measured at 76 CpG dinucleotides in the DMRs at the repetitive element *LINE-1* and four genes (*LINE-1*: 20 CpG sites; *MEST*: 7q32, 130486175–130506297, 12 CpG sites; *P16*: 9p21, 21967752–21995043, 18 CpG sites; *H19*: 11p15.5, 142575532–142578146, 14 CpG sites; and *GNAS*: 20q13.3, 58839740–58911195, 12 CpG sites; **Table 1**).

**Table 1:** Primer sequence of five genes.

Target gene	F	R	CpG	Position
*MEST*	GGGTTTAGAGGTATAAGAAAGAGGG	TTTCTAAAAACAACCAAACCCCTAC	1–17	chr7:130130648–130131063
*P16*	GTGGGTTTTAGTTTGTAGTTAAGGG	ATTATCTCCTCCTCCTCCTAACCTAA	1–35	chr9:21994026–21994435
*H16*	GAGATTTGAGGTGAATTTTAGGGA	CAAAACAAAATCCCCACAACC	1–20	chr11:2019635–2019930
*LINE1*	GGTGATTTTTGTATTTTTATTTGAGGT	CAAAAACAAACAAACCTCCTTAAACT	1–28	chr16:33760542–33761007
*GNAS*	GTTTTAGAGTTTTAGGGAAGGGGAG	ATCCCAAACTAACCAACTAAACCTC	1–19	chr20:57415713–57416072

### Statistical Analysis

All data were analyzed with peak picking spectra interpretation tools to generate the ratios of methyl CpG/total CpG. EpiTyper software (Sequenom, San Diego, CA, USA) was used to quantify the methylated fraction of all CpG units. Statistics 18.0 software (SPSS, Inc., Somers, NY, USA) was used to perform all the statistical analyses in this study. Pearson’s correlation coefficient test and analysis of variance followed by Dunnett’s *post hoct* test were used to compare the categorical variables and the differences in the mean values of continuous variables between the two groups. All tests were two-tailed. Principal component analysis (PCA) was performed to identify underlying factors. Kaiser–Meyer–Olkin (KMO) value and Bartlett’s test of sphericity were checked to confirm that the data were suitable for factor analysis. The criterion of eigenvalue >1.0 was applied to determine the number of factors retained. Items were included in the factor on which they loaded highest (minimum accepted 0.4).

## Results

The results of the sperm motility assessment and the levels of follicle-stimulating hormone (FSH), luteinizing hormone (LH), and testosterone (T) are shown in **Table 2**. Sperm motility was significantly lower in the nicotine-exposed (P = 0.0064) and the nicotine- and alcohol-exposed (P = 0.0008) groups than in the control group. FSH levels were higher in the nicotine-exposed group (P = 0.0026). Methylation of each CpG site and adjusted linear regression of alcohol and nicotine exposure are shown in **Suppl 1** and **Suppl 2**.

**Table 2:** Characteristics of all subjects.

Items mean (SD)	Neither nicotine nor alcohol exposed	Alcohol exposed only	Nicotine exposed only	Both nicotine and alcohol exposed	*P1*	*P2*	*P3*
N	48	16	16	63			
Age, years	30.13 (5.52)	36.56 (7.29)	39.60 (4.53)	38.68 (6.31)	**0.0004**	**<0.001**	**<0.001**
Marriage age, years,	24.35 (4.11)	21.07 (2.99)	27.92 (5.71)	24.69 (5.69)	**0.0064**	**0.0242**	0.7351
Body mass index	23.10 (4.02)	22.01 (2.05)	22.48 (2.71)	22.52 (2.42)	0.3186	0.6952	0.3651
Sexual absence, days	7.02 (5.70)	4.56 (4.29)	7.00 (7.48)	5.56 (8.47)	0.1196	0.9909	0.3039
Semen volume, ml,	2.96 (1.21)	2.50 (1.64)	2.90 (1.57)	2.52(1.39)	0.2366	0.8802	0.0869
Sperm concentration, ×10^6^/ml	65.47 (45.30)	65.40 (55.79)	55.31 (42.42)	58.23(47.94)	0.9958	0.4445	0.4213
Motility (moving forward), %	49.13 (12.20)	42.64 (17.97)	37.03 (20.34)	37.86(19.86)	0.1091	**0.0064**	**0.0008**
Vitality, %	68.50 (15.39)	63.00 (15.09)	62.38 (18.06)	67.30(16.20)	0.3733	0.3408	0.7583
LH, IU/L	5.32 (2.36)	4.25 (2.28)	6.02 (3.05)	4.94(3.97)	0.1181	0.3552	0.5603
T, IU/L	5.34 (1.96)	4.51 (2.47)	5.75 (1.81)	5.00(2.60)	0.1744	0.4735	0.4574
FSH, IU/L	5.05 (2.39)	4.16 (2.36)	7.87 (4.50)	5.69(5.74)	0.2003	**0.0026**	0.4734

P1: neither nicotine nor alcohol exposed vs alcohol exposed only; P2: neither nicotine- nor alcohol exposed vs nicotine exposed only; P3: neither nicotine nor alcohol exposed vs both nicotine and alcohol exposed. The bold represent p <0.05.

BMI, body mass index; FSH, Follicle-stimulating hormone, LH, luteinizing hormone; T, testosterone.

**Figure 1:**
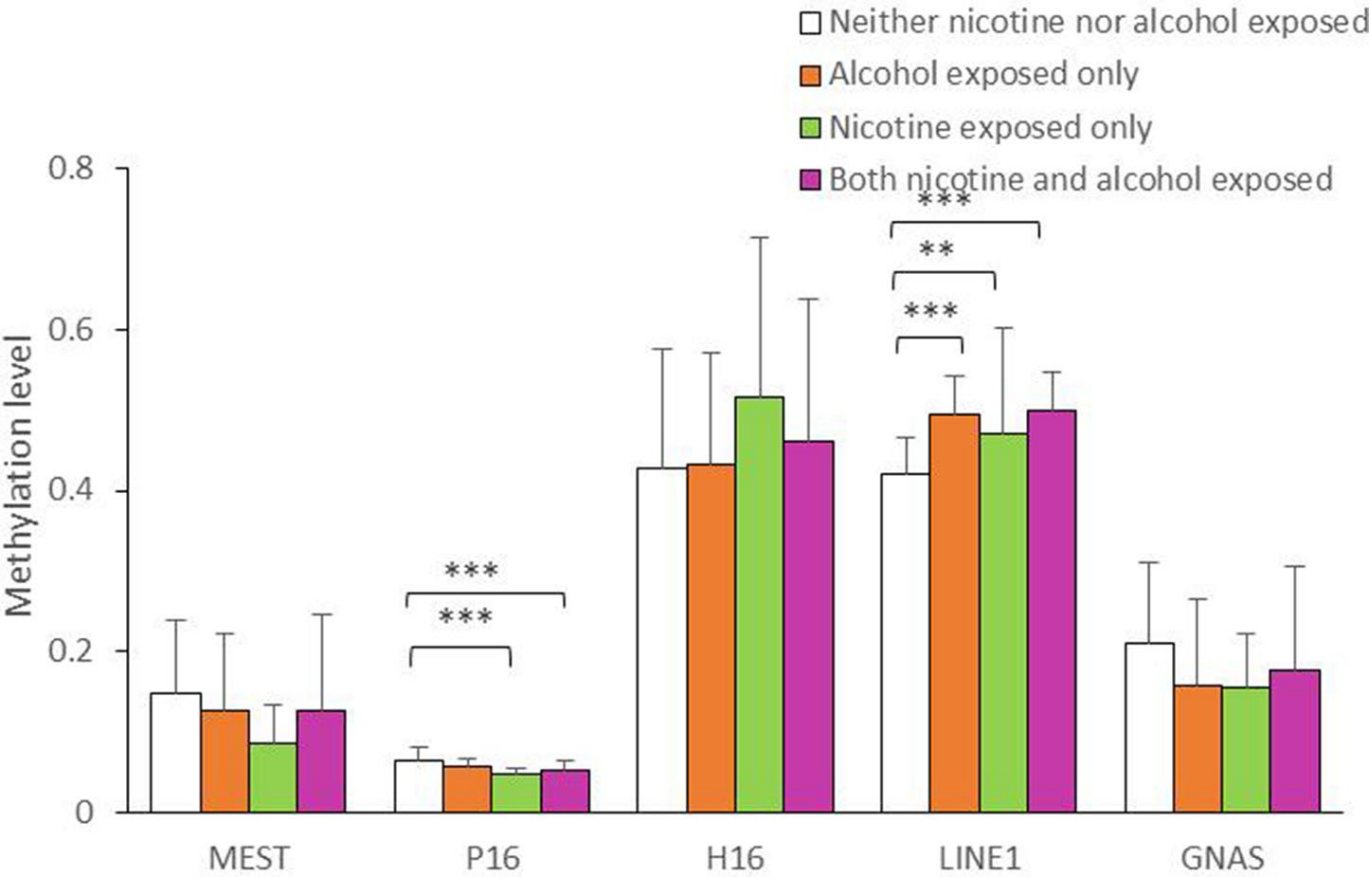
Average methylation level of the five genes in all the subjects. The comparison of average methylation levels at *MEST, P16, H19, LINE1*, and *GNAS* in human sperms of 143 minority male residents, respectively. Data are means ± SD. Statistically significant differences are represented with asterisks: **P < 0.01, ***P < 0.001. Not significant, P > 0.05.

**Figure 2:**
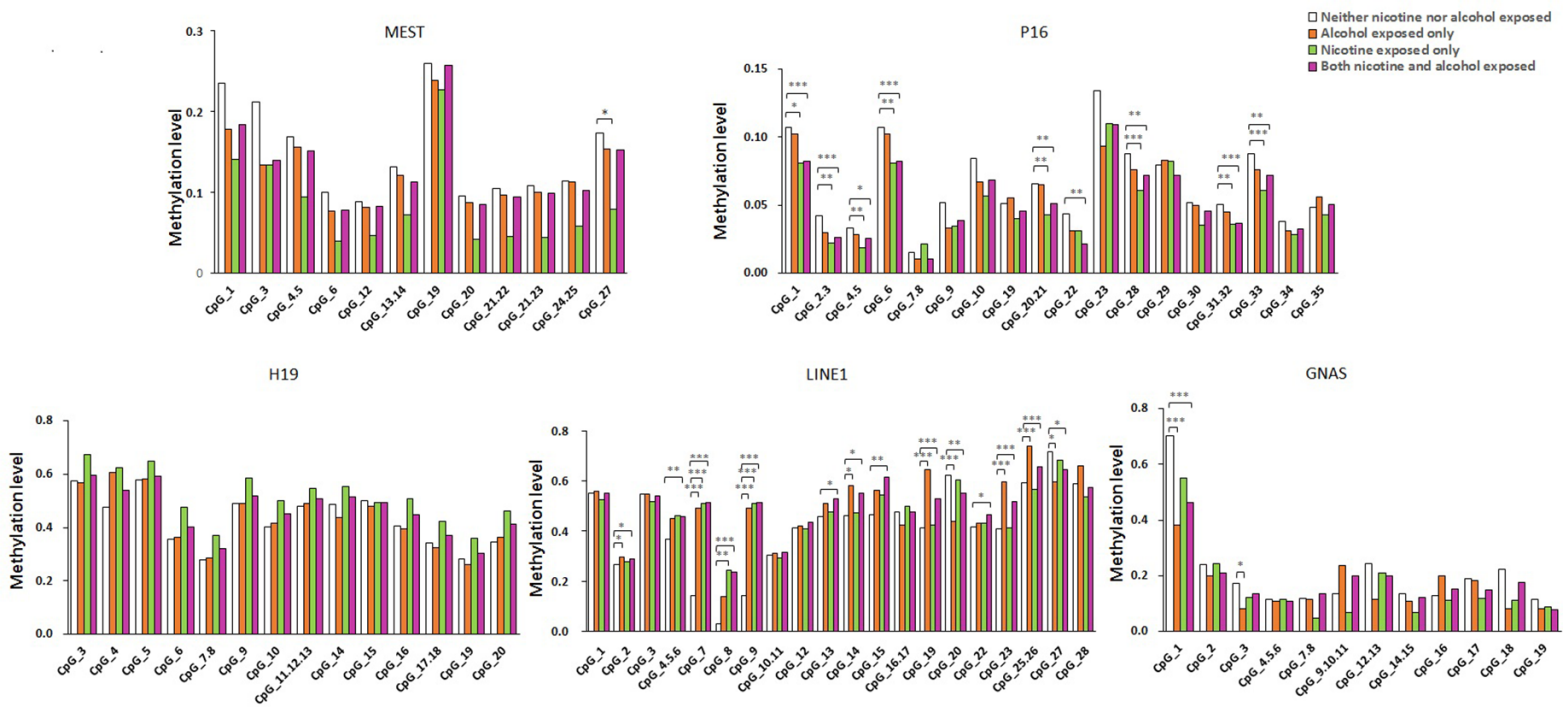
Methylation level of the five genes in all the subjects. The comparison of site-specific methylation levels at *MEST, P16, H19, LINE1*, and *GNAS* DMR of the neither nicotine- nor alcohol-exposed controls (N = 48) with alcohol-exposed only cases (N = 16), nicotine-exposed only cases (N = 16), both nicotine- and alcohol-exposed cases (N = 63), respectively. The data are the geometric mean ratios of methylation levels at each CpG site. *P < 0.05, **P < 0.01, ***P < 0.001. Not significant, P > 0.05

As shown in **Figure 1**, the average methylation levels of the repetitive element *LINE-1* and four assessed genes in sperm from 143 minority male residents were compared. In general, the methylation levels in the repetitive element *LINE-1* were higher in the three exposed groups (P < 0.001, P = 0.017, and P < 0.001, respectively) than in the control group, whereas methylation levels were lower in *P16* in the nicotine-exposed group and in the nicotine- and alcohol-exposed group after correction of multiple testing (P < 0.001, **Figure 1**). Individual CpG sites within the same gene showed similar trends in methylation level. Compared to the controls, the methylation levels of nine CpG sites in the repetitive element *LINE-1* (sites 2, 4.5.6, 7, 9, 14, 19, 20, 23, 25.26, and 27) were higher in the alcohol-exposed group, while the methylation levels of three CpG sites in the repetitive element *LINE-1* (sites 7, 8, and 9) were higher in the nicotine-exposed group, and the levels of 14 CpG sites were significantly higher in the nicotine- and alcohol-exposed group (**Figure 2**).

Among the imprinted genes, we found that the methylation levels of two CpG sites in the *GNAS* DMR (sites 1 and 3) were significantly lower in the alcohol-exposed and the nicotine- and alcohol-exposed groups, and only one site (site 11) in the *MEST* was lower in the nicotine-exposed group. However, the methylation levels of most CpGs in *H19* did not show obvious differences among the three groups (**Figure 2**).

Our results showed that the methylation levels of eight CpGs in *P16* in the nicotine-exposed and nicotine- and alcohol-exposed groups were lower than in the controls. In contrast, there were only one CpG sites with lower methylation levels in the alcohol-exposed group when compared to the control group (**Figure 2**).

**Figure 3:**
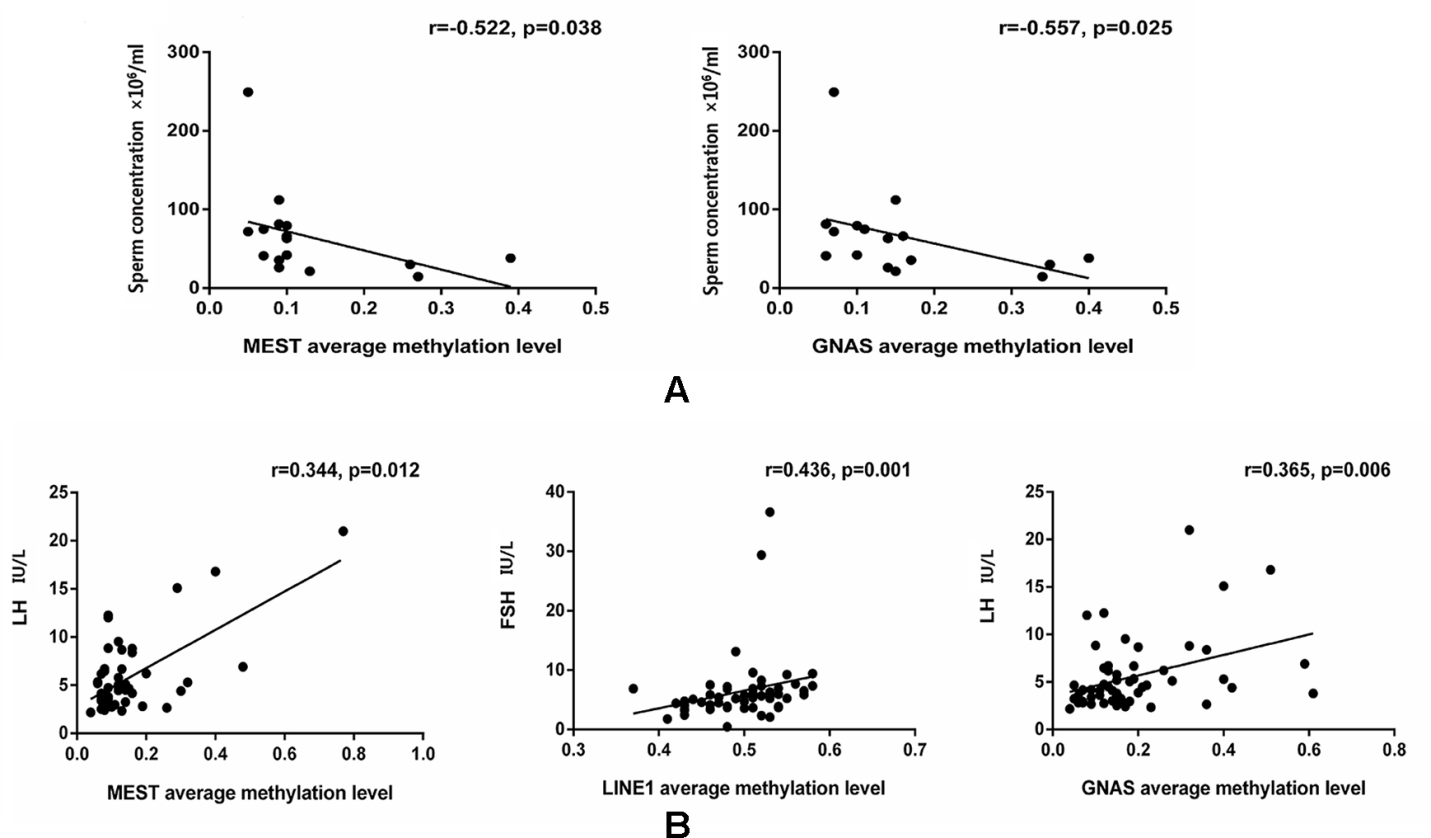
Comparison of the gene methylation levels and semen quality/hormone level. **(A)** The significant correlations between the average methylation levels, seminological parameters and hormones in the alcohol exposed only group. **(B)** The significant correlations between the average methylation levels, seminological parameters and hormones in the both nicotine and alcohol exposed group. The dots are the intersection points between the average methylation levels, seminological parameters and hormones.

**Figure 4:**
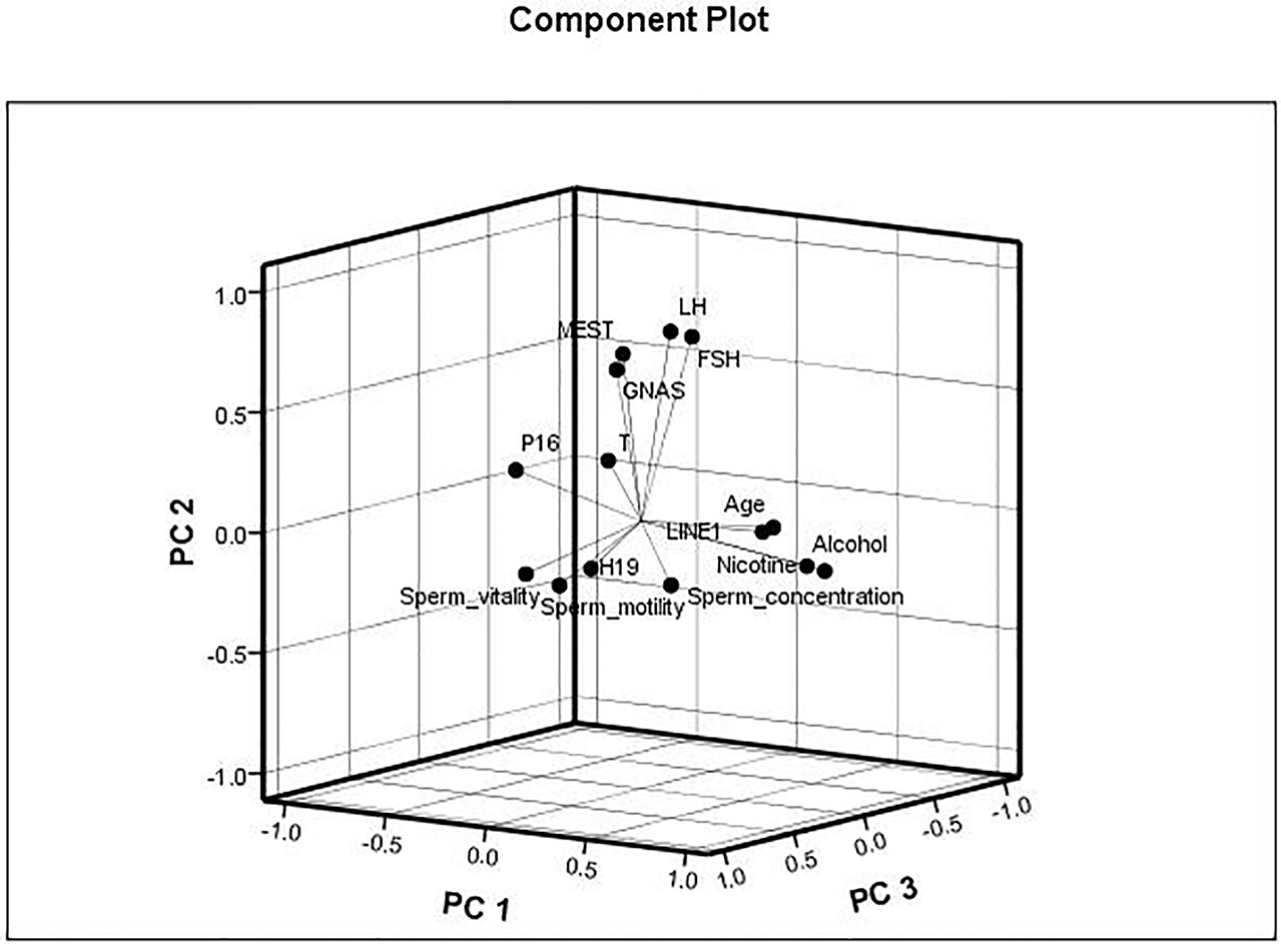
Multivariate correlation patterns. The Kaiser–Meyer–Olkin measure was 0.669, indicating that sufficient correlation existed between these variables to proceed with factor analysis. Three components were extracted by factor analysis using principal component analysis. Factor 1 labeled as “PC1” contains nicotine exposed, alcohol exposed, age, LINE1, P16, and sperm motility and had loadings of 0.852, 0.829, 0.678, 0.578, −0.516, and −0.403, respectively. This factor explained 20.0% of the total variance. Factor 2 labeled as “PC2” contains MEST, GNAS, follicle-stimulating hormone (FSH), and luteinizing hormone (LH) and had loadings of 0.672, 0.629, 0.775, and 0.789, respectively, explaining 17.5% of the total variance. Factor 3 labeled as “PC3” contains sperm vitality, H19, and sperm concentration and had loadings of 0.746, and 0.632, and −0.516, respectively, explaining 9.3% of the total variance.

The data were checked for normal distributions using the Shapiro–Wilk test. The correlations in methylation between loci were analyzed using the Pearson’s test. The methylation between *MEST* and *P16*, and *MEST* and *GNAS* were positively correlated, respectively (**Suppl 3**). The correlations between the average methylation levels and seminological parameters or hormones were analyzed using the Pearson’s (normal distributions) or Spearman’s correlation (abnormal distributions), respectively. Correlation tests for gene modulation levels and phenotypic indices showed that the average methylation levels of *MEST* and *GNAS* were inversely correlated with sperm concentration [r = −0.522 (P = 0.038) and r = −0.557 (P = 0.025), respectively; **Figure 3A**] in the alcohol-exposed group. The average methylation levels of *MEST* and *GNAS* were positively correlated with LH levels [r = 0.344 (P = 0.012) and r = 0.365 (P = 0.006), respectively], and the methylation of the repetitive element *LINE1* was positively correlated with FSH level (r = 0.436, P = 0.001; **Figure 3B**) in the nicotine- and alcohol-exposed group. However, no association between gene methylation and the phenotypic indices was observed in the nicotine-exposed group (P > 0.05, **Table 3**).The multivariate correlation pattern between the variables was investigated using PCA. The KMO measure was 0.669, indicating that sufficient correlation existed between these variables to proceed with factor analysis. Five components were extracted by factor analysis using PCA (**Suppl 4**). Variables located near each other such as MEST, GNAS, FSH, and LH were strongly correlated (**Figure 4**).

**Table 3:** Comparison of 5 gene methylation levels and semen quality/hormone level in all subjects.

	Sperm motility (% moving forward)		Concentration (106 spermatozoa/ml)		Sperm vitality (% alive)		FSH		LH		T	
	r	P	r	P	r	P	r	P	r	P	r	P
Neither nicotine nor alcohol Exposed												
MEST	−0.033	0.828	0.317	0.030	0.121	0.525	0.022	0.886	0.051	0.735	0.025	0.867
P16	−0.024	0.872	0.020	0.894	−0.119	0.524	0.211	0.154	−0.181	0.223	−0.006	0.970
H19	−0.276	0.061	−0.147	0.323	−0.373	0.039	0.263	0.078	0.177	0.240	0.027	0.857
LINE1	−0.101	0.496	−0.224	0.125	−0.171	0.357	0.071	0.635	−0.059	0.695	−0.040	0.789
GNAS	−0.171	0.246	−0.248	0.090	−0.199	0.283	0.081	0.586	0.120	0.420	0.072	0.628
Alcohol exposed only												
MEST	0.082	0.763	−0.522	**0.038**	0.024	0.955	−0.143	0.626	0.262	0.366	−0.389	0.169
P16	−0.114	0.674	−0.291	0.274	0.582	0.130	0.026	0.930	0.333	0.245	0.019	0.949
H19	−0.284	0.286	−0.350	0.184	0.306	0.461	−0.266	0.359	0.207	0.478	−0.089	0.761
LINE1	0.169	0.531	0.016	0.953	0.518	0.188	−0.082	0.780	0.113	0.701	−0.448	0.108
GNAS	−0.054	0.843	−0.557	**0.025**	−0.005	0.986	−0.207	0.478	0.176	0.547	0.044	0.881
Nicotine exposed only												
MEST	0.041	0.884	−0.474	0.075	−0.333	0.347	0.088	0.745	−0.044	0.871	−0.362	0.169
P16	−0.354	0.196	−0.104	0.712	0.034	0.925	−0.108	0.692	−0.327	0.216	0.219	0.415
H19	−0.134	0.633	−0.427	0.113	0.604	0.064	−0.076	0.778	−0.071	0.795	−0.062	0.820
LINE1	0.056	0.849	0.041	0.890	−0.240	0.534	0.046	0.869	0.175	0.532	−0.122	0.664
GNAS	0.382	0.160	0.062	0.825	−0.544	0.104	0.209	0.438	0.482	0.058	−0.358	0.173
Both nicotine and alcohol exposed												
MEST	−0.062	0.648	−0.196	0.144	−0.023	0.889	0.241	0.082	0.344	**0.012**	0.016	0.910
P16	0.064	0.631	0.072	0.589	−0.050	0.761	0.097	0.481	0.019	0.892	−0.074	0.593
H19	−0.140	0.295	−0.103	0.441	−0.065	0.685	−0.059	0.673	0.024	0.864	0.184	0.186
LINE1	−0.037	0.780	−0.077	0.561	−0.067	0.676	0.436	**0.001**	0.173	0.206	0.168	0.221
GNAS	−0.187	0.159	−0.218	0.101	−0.087	0.593	0.194	0.156	0.365	**0.006**	0.066	0.630

The bold represent p<0.05.

## Discussion

In this study, we observed that alcohol and nicotine exposure altered sperm cell quality, which may be related to the methylation levels of *MEST* and *GNAS*. The methylation levels of *MEST*, *GNAS*, and the repetitive element *LINE1* were significantly associated with sperm concentration and FSH and LH levels.

Recent studies have shown that tobacco use and alcohol consumption may increase the risk of global aberrant DNA methylation (Hamid et al., 2009; Semmler et al., 2015). Aberrant methylation of *LINE-1* has been reported in many recent studies on aging (Cho et al., 2015) and cancer (Pattamadilok et al., 2008; Suzuki et al., 2013; Suter et al., 2004), and *LINE-1* methylation status could reflect the influence of environmental conditions or lifestyle habits on the genome (Schernhammer et al., 2010). High tobacco use might lead to a high risk of *LINE-1* hypermethylation-related cancers in men (Andreotti et al., 2014; Karami et al., 2015). To the best of our knowledge, our study showed, for the first time, that hypermethylation of the *LINE-1* gene in sperm was associated with alcohol and nicotine exposure. However, the association between *LINE-1* methylation level and alcohol and tobacco exposure in sperm needs to be further explored in future studies.

In our study, compared to the control group, hypomethylation of *P16* was observed in the tobacco-exposed group. In addition, we found that *GNAS* methylation was decreased in the alcohol-exposed group, and *P16* methylation was decreased in the nicotine- and alcohol-exposed group. Previous studies have suggested that chronic paternal alcohol exposure might contribute to mental deficits in offspring *via* abnormal methylation of imprinted genes (such as *H19* and *Peg3*) in sperm (Liang et al., 2014) and that methylation levels could be easily modified by air pollution, heavy metals, and other environmental factors, both *in vivo* and *in vitro* (Baccarelli and Bollati, 2009; Haggarty, 2013). An association between nicotine/alcohol exposure and methylation has been demonstrated in pregnant women (Barua and Junaid, 2015; Lee et al., 2015). In this study, we studied the influence of nicotine/alcohol exposure in male residents from Guizhou province, a population with a low fertility rate. The relationship between nicotine/alcohol exposure and the methylation of these five genes in male residents of Guizhou has not been previously reported. Abnormal DNA methylation in spermatozoa seems to be involved in environmental factor-induced transgenerational disruptive spermatogenesis (Anway et al., 2005; Anway et al., 2006). The impact of environmental factors on the epigenetic phenotype might affect offspring through abnormal spermatozoa methylation (Anway and Skinner, 2006). Some evidence has suggested that abnormal DNA methylation of imprinted genes may be associated with spermatogenesis failure (Boissonnas et al., 2010), and an observable decrease in the concentration of sperm was reported in patients with *H19* hypomethylation (Stouder et al., 2011). In addition, aberrant *MEST* DNA methylation has been shown to be significantly associated with increased FSH levels (Klaver et al., 2013). However, we found that alcohol exposure altered sperm cell quality and was related to the hypomethylation of *MEST* and *GNAS*. *MEST* and *GNAS* methylation levels were significantly associated with increased LH levels, and *LINE1* methylation was significantly associated with increased FSH levels. However, further studies are needed to explore the mechanisms underlying the association between chronic nicotine and alcohol exposure and aberration methylation of *MEST*, *GNAS*, and *LINE1* with sperm quality and abnormal FSH and LH levels.

Our study has several limitations. First, our study had a small sample size, which restricts the generalizability of our results. Therefore, our results must be verified in larger cohorts using different techniques. In addition, further studies are needed to explore how changes in methylation due to smoking and drinking affect fertility.

In conclusion, our results show that the different methylation levels of four genes, *MEST*, *P16*, *LINE-1*, and *GNAS*, alter the sperm cells of patients who consume alcohol and use nicotine. Both smoking and drinking impair sperm/semen quality and hormone levels. Thus, methylation of *MEST*, *GNAS*, and *LINE1* may be associated with sperm concentration and FSH and LH levels.

## Ethics Statement

Our study was approved by the Ethics Committee of Shanghai Institute of Planned Parenthood Research (SIPPR) and the local approval of Guizhou province was not required.

## Author Contributions

Designed and coordinated the study: WZ, AZ, HS, JD. Performed the experiments: FS, ZZ, YS, JX, ML. Analyzed the data: XX, JL, XS, MM, JD. Contributed reagents/materials/analysis tools: YY, FS, BW. Wrote the paper: WZ, FS, JD. Helped in critical revision of the manuscript for important intellectual content: DJ, XH, ML.

## Conflict of Interest

The authors declare that the research was conducted in the absence of any commercial or financial relationships that could be construed as a potential conflict of interest.
