# Supplementary material for: Association of Sperm Methylation at LINE-1, Four Candidate Genes, and Nicotine/Alcohol Exposure With the Risk of Infertility
Source: Front Genet. 2019 Oct 18;10:1001. doi: 10.3389/fgene.2019.01001 (PMC6813923; doi:10.3389/fgene.2019.01001)
Supplement: Supplementary file 1 [file Table_1.docx]

Suppl 1. Methylation of each CpG sites(GM ratio (std))

| Items | Neither Nicotine nor Alcohol Exposed | Alcohol Exposed only | Nicotine Exposed only | Both Nicotine and Alcohol Exposed |
| --- | --- | --- | --- | --- |
| MEST_CpG_1 | 0.20(1.89) | 0.15(1.89) | 0.13(1.74) | 0.15084(2.00140) |
| MEST_CpG_3 | 0.18(2.44) | 0.13(2.40) | 0.09(3.07) | 0.13(2.15) |
| MEST_CpG_4.5 | 0.14(1.95) | 0.12(2.01) | 0.09(1.73) | 0.12(2.21) |
| MEST_CpG_6 | 0.09(2.48) | 0.06(2.66) | 0.04(2.76) | 0.06(2.79) |
| MEST_CpG_12 | 0.08(1.68) | 0.07(1.68) | 0.05(1.42) | 0.07(2.01) |
| MEST_CpG_13.14 | 0.12(1.94) | 0.09(2.07) | 0.07(1.72) | 0.09(2.23) |
| MEST_CpG_19 | 0.26(1.35) | 0.23(1.30) | 0.23(1.33) | 0.26(1.34) |
| MEST_CpG_20 | 0.07(2.35) | 0.06(2.27) | 0.04(2.30) | 0.06(2.31) |
| MEST_CpG_21.22 | 0.07(2.58) | 0.07(2.22) | 0.04(2.18) | 0.06(2.57) |
| MEST_CpG_21.23 | 0.09(2.26) | 0.06(2.62) | 0.04(1.94) | 0.07(2.46) |
| MEST_CpG_24.25 | 0.09(2.01) | 0.08(2.07 ) | 0.05(1.88) | 0.07(2.48) |
| MEST_CpG_27 | 0.15(1.87) | 0.12(2.21) | 0.07(2.20) | 0.13(1.85) |
| P16_CpG_1 | 0.10(1.34) | 0.10(1.22) | 0.08(1.49) | 0.08(1.54) |
| P16_CpG_2.3 | 0.04(1.75) | 0.03(1.71) | 0.02(1.62) | 0.03(1.64) |
| P16_CpG_4.5 | 0.03(1.68) | 0.02(1.71) | 0.02(1.44) | 0.021.72) |
| P16_CpG_6 | 0.10(1.34) | 0.10(1.22) | 0.08(1.49) | 0.08(1.54) |
| P16_CpG_7.8 | 0.03(2.07) | 0.01(1.59) | 0.02(2.35) | 0.02(1.78) |
| P16_CpG_9 | 0.04(2.09) | 0.03(1.84) | 0.03(1.88) | 0.03(1.99) |
| P16_CpG_10 | 0.07(1.81) | 0.06(1.82) | 0.05(2.08) | 0.06(2.09) |
| P16_CpG_19 | 0.05(1.42) | 0.05(1.37) | 0.04(1.43) | 0.05(1.36) |
| P16_CpG_20.21 | 0.06(1.62) | 0.06(1.38) | 0.04(1.78) | 0.06(1.43) |
| P16_CpG_22 | 0.05(2.19) | 0.03(2.03) | 0.05(2.44) | 0.03(2.02) |
| P16_CpG_23 | 0.13(1.75) | 0.09(1.57) | 0.10(1.93 ) | 0.10(1.91) |
| P16_CpG_28 | 0.09(1.30) | 0.08(1.16) | 0.06(1.34) | 0.07(1.42) |
| P16_CpG_29 | 0.07(1.68) | 0.08(1.47) | 0.06(2.31) | 0.06(1.74) |
| P16_CpG_30 | 0.05(1.61) | 0.04(1.81) | 0.03(1.77) | 0.04(1.98) |
| P16_CpG_31.32 | 0.05(1.50) | 0.04(1.38) | 0.04(1.48) | 0.04(1.61) |
| P16_CpG_33 | 0.09(1.30) | 0.08(1.16) | 0.06(1.34) | 0.07(1.42) |
| P16_CpG_34 | 0.04(1.75) | 0.03(1.48) | 0.03(1.74) | 0.03(1.82) |
| P16_CpG_35 | 0.05(1.49) | 0.05(1.46) | 0.04(1.39) | 0.05(1.52) |
| H19_CpG_3 | 0.56(1.32) | 0.55(1.27) | 0.63(1.48) | 0.60(1.41) |
| H19_CpG_4 | 0.52(2.11) | 0.62(1.33) | 0.69(1.34) | 0.66(1.33) |
| H19_CpG_5 | 0.57(1.32) | 0.56(1.32) | 0.62(1.43) | 0.58(1.41) |
| H19_CpG_6 | 0.34(1.49) | 0.34(1.57) | 0.42(1.73) | 0.38(1.76) |
| H19_CpG_7.8 | 0.25(1.64) | 0.26(1.63) | 0.31(1.90) | 0.29(1.71) |
| H19_CpG_9 | 0.47(1.42) | 0.46(1.43) | 0.53(1.61) | 0.51(1.46) |
| H19_CpG_10 | 0.38(1.47) | 0.39(1.549) | 0.45(1.67) | 0.44(1.54) |
| H19_CpG_11.12.13 | 0.48(1.27) | 0.48(1.28) | 0.53(1.29) | 0.51(1.36) |
| H19_CpG_14 | 0.48(1.48) | 0.40(1.65 | 0.51(1.55) | 0.50(1.46) |
| H19_CpG_15 | 0.47(1.49) | 0.45(1.47) | 0.45(1.58) | 0.47(1.53) |
| H19_CpG_16 | 0.38(1.48) | 0.37(1.52) | 0.46(1.65) | 0.43(1.54) |
| H19_CpG_17.18 | 0.32(1.59) | 0.30(1.57) | 0.37(1.72) | 0.345(1.63) |
| H19_CpG_19 | 0.27(1.44) | 0.25(1.46) | 0.32(1.69) | 0.28(1.58) |
| H19_CpG_20 | 0.33(1.53) | 0.34(1.53) | 0.41(1.71) | 0.37(1.77) |
| LINE-1_CpG_1 | 0.55(1.07) | 0.56(1.07) | 0.56(1.03) | 0.55(1.08) |
| LINE-1_CpG_2 | 0.26(1.16) | 0.29(1.08) | 0.30(1.08) | 0.29(1.13) |
| LINE-1_CpG_3 | 0.55(1.09) | 0.55(1.11) | 0.55(1.08) | 0.54(1.12) |
| LINE-1_CpG_4.5.6 | 0.46(1.11) | 0.48(1.12) | 0.49(1.07) | 0.46(1.11520) |
| LINE-1_CpG_7 | 0.57(1.07) | 0.61(1.08) | 0.63(1.06) | 0.62(1.12) |
| LINE-1_CpG_8 | 0.46(1.06) | 0.44(1.14) | 0.43(1.17) | 0.43(1.22) |
| LINE-1_CpG_9 | 0.57(1.07 ) | 0.61(1.08) | 0.63(1.06) | 0.62(1.12) |
| LINE-1_CpG_10.11 | 0.31(1.07) | 0.31(1.05) | 0.31(1.06) | 0.31(1.07) |
| LINE-1_CpG_12 | 0.41(1.10) | 0.42(1.08) | 0.43(1.07) | 0.43(1.10) |
| LINE-1_CpG_13 | 0.47(1.31) | 0.50(1.18) | 0.50(1.17) | 0.53(1.24) |
| LINE-1_CpG_14 | 0.48(1.32) | 0.66(1.15) | 0.50(1.17) | 0.56(1.24) |
| LINE-1_CpG_15 | 0.63(1.16) | 0.55(1.20) | 0.67(1.09) | 0.62(1.20) |
| LINE-1_CpG_16.17 | 0.47(1.12) | 0.42(1.16) | 0.53(1.14 ) | 0.48(1.21) |
| LINE-1_CpG_19 | 0.41(1.17) | 0.64(1.08) | 0.45(1.08) | 0.52(1.23) |
| LINE-1_CpG_20 | 0.62(1.10) | 0.44(1.06) | 0.64(1.05) | 0.54(1.25) |
| LINE-1_CpG_22 | 0.41(1.09) | 0.43(1.07) | 0.46(1.16) | 0.46(1.22) |
| LINE-1_CpG_23 | 0.40(1.28) | 0.60(1.09) | 0.44(1.09) | 0.52(1.21) |
| LINE-1_CpG_25.26 | 0.59(1.10) | 0.74(1.06) | 0.60(1.08) | 0.65(1.13) |
| LINE-1_CpG_27 | 0.72(1.07) | 0.60(1.09) | 0.73(1.09) | 0.59(1.92) |
| LINE-1_CpG_28 | 0.59(1.09) | 0.66(1.10) | 0.52(1.75) | 0.54(1.54) |
| Primer18_CpG_1 | 0.68(1.29) | 0.31(2.49) | 0.56(1.49) | 0.40(2.76) |
| Primer18_CpG_2 | 0.17(2.84) | 0.18(1.71) | 0.18(2.89) | 0.18(2.40) |
| Primer18_CpG_3 | 0.15(1.64) | 0.12(2.62) | 0.12(1.66) | 0.13(2.11) |
| Primer18_CpG_4.5.6 | 0.13(1.96) | 0.07(2.35) | 0.13(2.10) | 0.10(2.49) |
| Primer18_CpG_7.8 | 0.08(2.35) | 0.09(2.22) | 0.05(1.74) | 0.09(2.56) |
| Primer18_CpG_9.10.11 | 0.10(2.17) | 0.19(2.05) | 0.07(1.78) | 0.14(2.43) |
| Primer18_CpG_12.13 | 0.21(1.68) | 0.09(2.22) | 0.18(1.75) | 0.14(2.45) |
| Primer18_CpG_14.15 | 0.10(2.15) | 0.07(2.76 ) | 0.07(1.78) | 0.08(2.78) |
| Primer18_CpG_16 | 0.08(2.59) | 0.18(1.71) | 0.08(2.44) | 0.12(2.36) |
| Primer18_CpG_17 | 0.16(1.65) | 0.12(4.06) | 0.13(1.54) | 0.12(3.06) |
| Primer18_CpG_18 | 0.11(4.02) | 0.12(2.62) | 0.09(2.92) | 0.14(3.50) |
| Primer18_CpG_19 | 0.13(1.96) | 0.12(6.01 ) | 0.12(1.94) | 0.11(3.06) |
