# Supplementary material for: Association of Sperm Methylation at LINE-1, Four Candidate Genes, and Nicotine/Alcohol Exposure With the Risk of Infertility
Source: Front Genet. 2019 Oct 18;10:1001. doi: 10.3389/fgene.2019.01001 (PMC6813923; doi:10.3389/fgene.2019.01001)
Supplement: Supplementary file 2 [file Table_2.docx]

Suppl 2. Adjust linear regression of alcohol and nicotine exposure and methylation of each CpG sites

| Items | Neither Nicotine nor Alcohol Exposed | Alcohol Exposed only | | Nicotine Exposed only | | Both Nicotine and Alcohol Exposed | |
| --- | --- | --- | --- | --- | --- | --- | --- |
|  |  | Adjust β | *p* | Adjust β | *p* | Adjust β | *p* |
| MEST_CpG_1 | ref | -0.282 | 0.197 | -0.568 | 0.008 | -0.301 | 0.072 |
| MEST_CpG_3 | ref | -0.305 | 0.352 | -0.906 | 0.004 | -0.419 | 0.086 |
| MEST_CpG_4.5 | ref | -0.163 | 0.502 | -0.679 | 0.006 | -0.329 | 0.078 |
| MEST_CpG_6 | ref | -0.772 | 0.044 | -1.398 | 0.000 | -0.742 | 0.011 |
| MEST_CpG_12 | ref | -0.154 | 0.444 | -0.670 | 0.001 | -0.296 | 0.056 |
| MEST_CpG_13.14 | ref | -0.272 | 0.278 | -0.705 | 0.004 | -0.277 | 0.151 |
| MEST_CpG_19 | ref | -0.095 | 0.330 | -0.141 | 0.134 | 0.016 | 0.830 |
| MEST_CpG_20 | ref | -0.236 | 0.423 | -0.905 | 0.004 | -0.281 | 0.215 |
| MEST_CpG_21.22 | ref | -0.090 | 0.768 | -0.954 | 0.002 | -0.298 | 0.201 |
| MEST_CpG_21.23 | ref | -0.410 | 0.165 | -1.077 | 0.001 | -0.370 | 0.115 |
| MEST_CpG_24.25 | ref | -0.071 | 0.791 | -0.843 | 0.001 | -0.425 | 0.038 |
| MEST_CpG_27 | ref | -0.159 | 0.484 | -0.846 | 0.000 | -0.041 | 0.814 |
| P16_CpG_1 | ref | -0.001 | 0.994 | -0.171 | 0.138 | -0.198 | 0.030 |
| P16_CpG_2.3 | ref | -0.256 | 0.172 | -0.486 | 0.010 | -0.287 | 0.050 |
| P16_CpG_4.5 | ref | -0.095 | 0.600 | -0.431 | 0.020 | -0.210 | 0.140 |
| P16_CpG_6 | ref | -0.001 | 0.994 | -0.171 | 0.138 | -0.198 | 0.030 |
| P16_CpG_7.8 | ref | -0.744 | 0.021 | -0.484 | 0.108 | -0.516 | 0.066 |
| P16_CpG_9 | ref | -0.387 | 0.099 | -0.183 | 0.442 | -0.140 | 0.446 |
| P16_CpG_10 | ref | -0.485 | 0.036 | -0.646 | 0.004 | -0.398 | 0.025 |
| P16_CpG_19 | ref | 0.016 | 0.887 | -0.279 | 0.010 | -0.087 | 0.301 |
| P16_CpG_20.21 | ref | -0.001 | 0.996 | -0.485 | 0.001 | -0.237 | 0.036 |
| P16_CpG_22 | ref | -0.774 | 0.011 | -0.001 | 0.999 | -0.717 | 0.008 |
| P16_CpG_23 | ref | -0.602 | 0.007 | -0.326 | 0.124 | -0.450 | 0.007 |
| P16_CpG_28 | ref | -0.034 | 0.746 | -0.248 | 0.020 | -0.151 | 0.064 |
| P16_CpG_29 | ref | -0.039 | 0.826 | -0.606 | 0.001 | -0.248 | 0.068 |
| P16_CpG_30 | ref | -0.171 | 0.417 | -0.369 | 0.087 | -0.280 | 0.090 |
| P16_CpG_31.32 | ref | -0.347 | 0.018 | -0.497 | 0.001 | -0.561 | <.0001 |
| P16_CpG_33 | ref | -0.034 | 0.746 | -0.248 | 0.020 | -0.151 | 0.064 |
| P16_CpG_34 | ref | -0.249 | 0.189 | -0.493 | 0.008 | -0.387 | 0.008 |
| P16_CpG_35 | ref | 0.055 | 0.695 | -0.135 | 0.325 | -0.072 | 0.505 |
| H19_CpG_3 | ref | -0.022 | 0.836 | 0.109 | 0.295 | 0.044 | 0.590 |
| H19_CpG_4 | ref | 0.258 | 0.170 | 0.417 | 0.029 | 0.371 | 0.016 |
| H19_CpG_5 | ref | -0.029 | 0.788 | 0.077 | 0.457 | 0.009 | 0.909 |
| H19_CpG_6 | ref | 0.025 | 0.891 | 0.184 | 0.293 | 0.122 | 0.375 |
| H19_CpG_7.8 | ref | 0.008 | 0.964 | 0.100 | 0.577 | 0.098 | 0.487 |
| H19_CpG_9 | ref | -0.001 | 0.991 | 0.102 | 0.423 | 0.081 | 0.423 |
| H19_CpG_10 | ref | 0.013 | 0.928 | 0.085 | 0.557 | 0.095 | 0.403 |
| H19_CpG_11.12.13 | ref | 0.047 | 0.635 | 0.111 | 0.254 | 0.079 | 0.303 |
| H19_CpG_14 | ref | -0.158 | 0.268 | 0.012 | 0.929 | 0.035 | 0.751 |
| H19_CpG_15 | ref | -0.010 | 0.947 | -0.079 | 0.584 | 0.024 | 0.829 |
| H19_CpG_16 | ref | -0.039 | 0.792 | 0.116 | 0.426 | 0.082 | 0.473 |
| H19_CpG_17.18 | ref | -0.029 | 0.867 | 0.098 | 0.556 | 0.067 | 0.608 |
| H19_CpG_19 | ref | -0.075 | 0.614 | 0.125 | 0.387 | 0.046 | 0.688 |
| H19_CpG_20 | ref | 0.095 | 0.600 | 0.200 | 0.254 | 0.155 | 0.264 |
| LINE-1_CpG_1 | ref | 0.023 | 0.277 | -0.003 | 0.885 | -0.017 | 0.295 |
| LINE-1_CpG_2 | ref | 0.123 | 0.005 | 0.107 | 0.014 | 0.075 | 0.027 |
| LINE-1_CpG_3 | ref | -0.011 | 0.725 | -0.054 | 0.088 | -0.063 | 0.011 |
| LINE-1_CpG_4.5.6 | ref | 0.054 | 0.143 | 0.031 | 0.373 | -0.008 | 0.780 |
| LINE-1_CpG_7 | ref | 0.053 | 0.234 | 0.062 | 0.176 | 0.069 | 0.066 |
| LINE-1_CpG_8 | ref | -0.227 | 0.155 | -0.262 | 0.065 | -0.231 | 0.079 |
| LINE-1_CpG_9 | ref | 0.053 | 0.234 | 0.062 | 0.176 | 0.069 | 0.066 |
| LINE-1_CpG_10.11 | ref | -0.001 | 0.949 | -0.008 | 0.707 | -0.001 | 0.951 |
| LINE-1_CpG_12 | ref | -0.001 | 0.979 | 0.008 | 0.792 | 0.013 | 0.601 |
| LINE-1_CpG_13 | ref | 0.042 | 0.602 | 0.046 | 0.560 | 0.080 | 0.198 |
| LINE-1_CpG_14 | ref | 0.333 | 0.000 | -0.003 | 0.975 | 0.138 | 0.026 |
| LINE-1_CpG_15 | ref | -0.180 | 0.003 | -0.019 | 0.753 | -0.115 | 0.015 |
| LINE-1_CpG_16.17 | ref | -0.142 | 0.005 | 0.023 | 0.648 | -0.076 | 0.052 |
| LINE-1_CpG_19 | ref | 0.468 | <.0001 | 0.113 | 0.045 | 0.270 | <.0001 |
| LINE-1_CpG_20 | ref | -0.362 | <.0001 | -0.047 | 0.345 | -0.219 | <.0001 |
| LINE-1_CpG_22 | ref | 0.007 | 0.884 | 0.047 | 0.330 | 0.023 | 0.532 |
| LINE-1_CpG_23 | ref | 0.360 | <.0001 | 0.064 | 0.325 | 0.215 | <.0001 |
| LINE-1_CpG_25.26 | ref | 0.209 | <.0001 | 0.023 | 0.508 | 0.084 | 0.002 |
| LINE-1_CpG_27 | ref | -0.114 | 0.478 | 0.030 | 0.848 | -0.173 | 0.160 |
| LINE-1_CpG_28 | ref | 0.162 | 0.118 | -0.104 | 0.307 | 0.010 | 0.894 |
| Primer18_CpG_1 | ref | -0.758 | 0.006 | -0.219 | 0.406 | -0.507 | 0.015 |
| Primer18_CpG_2 | ref | 0.042 | 0.892 | -0.375 | 0.242 | -0.061 | 0.805 |
| Primer18_CpG_3 | ref | -0.223 | 0.417 | -0.499 | 0.026 | -0.338 | 0.070 |
| Primer18_CpG_4.5.6 | ref | -0.983 | 0.002 | -0.172 | 0.587 | -0.779 | 0.002 |
| Primer18_CpG_7.8 | ref | -0.109 | 0.715 | -0.971 | 0.001 | -0.287 | 0.216 |
| Primer18_CpG_9.10.11 | ref | 0.479 | 0.084 | -0.511 | 0.059 | 0.335 | 0.113 |
| Primer18_CpG_12.13 | ref | -1.056 | <.0001 | -0.600 | 0.015 | -0.769 | 0.000 |
| Primer18_CpG_14.15 | ref | -0.397 | 0.208 | -0.681 | 0.028 | -0.428 | 0.081 |
| Primer18_CpG_16 | ref | 0.779 | 0.009 | -0.429 | 0.136 | 0.269 | 0.232 |
| Primer18_CpG_17 | ref | -0.456 | 0.212 | -0.516 | 0.105 | -0.631 | 0.015 |
| Primer18_CpG_18 | ref | -0.037 | 0.944 | -0.945 | 0.058 | -0.307 | 0.411 |
| Primer18_CpG_19 | ref | -0.193 | 0.730 | 0.085 | 0.840 | -0.267 | 0.442 |

Adjust for age, married age, BMI, Motility, FSH, LH, T
