# Supplementary material for: Association of Sperm Methylation at LINE-1, Four Candidate Genes, and Nicotine/Alcohol Exposure With the Risk of Infertility
Source: Front Genet. 2019 Oct 18;10:1001. doi: 10.3389/fgene.2019.01001 (PMC6813923; doi:10.3389/fgene.2019.01001)
Supplement: Supplementary file 3 [file Table_3.docx]

Suppl 3 Correlations in methylation between loci

|  | MEST | |  | P16 | |  | | H19 | | |  | | LINE1 | | |
| --- | --- | --- | --- | --- | --- | --- | --- | --- | --- | --- | --- | --- | --- | --- | --- |
|  | r | *p* |  | r | *p* | |  | | r | *p* | |  | | r | *p* |
| P16 | 0.210 | **0.012** |  |  |  | |  | |  |  | |  | |  |  |
| H19 | -0.090 | 0.286 |  | 0.055 | 0.513 | |  | |  |  | |  | |  |  |
| LINE1 | 0.055 | 0.515 |  | -0.157 | 0.060 | |  | | 0.092 | 0.276 | |  | |  |  |
| GNAS | 0.447 | **<0.001** |  | 0.078 | 0.357 | |  | | -0.084 | 0.318 | |  | | 0.006 | 0.940 |
