# Supplementary material for: Association of Sperm Methylation at LINE-1, Four Candidate Genes, and Nicotine/Alcohol Exposure With the Risk of Infertility
Source: Front Genet. 2019 Oct 18;10:1001. doi: 10.3389/fgene.2019.01001 (PMC6813923; doi:10.3389/fgene.2019.01001)
Supplement: Supplementary file 4 [file Table_4.docx]

Suppl 4 Factor loadings for five factors

|  | Factor | | | | |
| --- | --- | --- | --- | --- | --- |
|  | 1 | 2 | 3 | 4 | 5 |
| Nicotine exposed | .852 |  |  |  |  |
| Alcohol exposed | .829 |  |  |  |  |
| Age | .678 |  |  |  |  |
| MEST |  | .672 |  |  | .448 |
| P16 | -.516 |  |  |  |  |
| H19 |  |  | .632 |  |  |
| LINE1 | .578 |  |  |  |  |
| GNAS |  | .629 |  |  |  |
| Sperm concentration |  |  | -.516 | .504 |  |
| Sperm motility | -.403 |  |  | .591 |  |
| Sperm vitality |  |  | .746 |  |  |
| FSH |  | .775 |  |  |  |
| LH |  | .789 |  |  |  |
| T |  |  |  |  | -.755 |
